# Supplementary material for: Single Particle Dynamics of Protein Aggregation and Disaggregation in the Presence of the sHsp Proteins IbpAB
Source: Biochemistry. 2025 Sep 26;64(19):4181–95. doi: 10.1021/acs.biochem.5c00312 (PMC12509322; doi:10.1021/acs.biochem.5c00312)
Supplement: Supplementary file 1 [file bi5c00312_si_001.pdf]

SUPPORTING INFORMATION

Single particle dynamics of protein aggregation and disaggregation in the presence of the sHsp proteins IbpAB

Andrew Roth[1], YuChen Yang[1], Jason Puchalla[2], and Hays S. Rye[1]*

[1] *Department of Biochemistry and Biophysics, Texas A&M University, College Station, Texas, 77845, USA*

[2] *Department of Physics, Princeton University, Princeton, NJ, 08544, USA*

Correspondence should be addressed to H. S. R. (haysrye@tamu.edu)

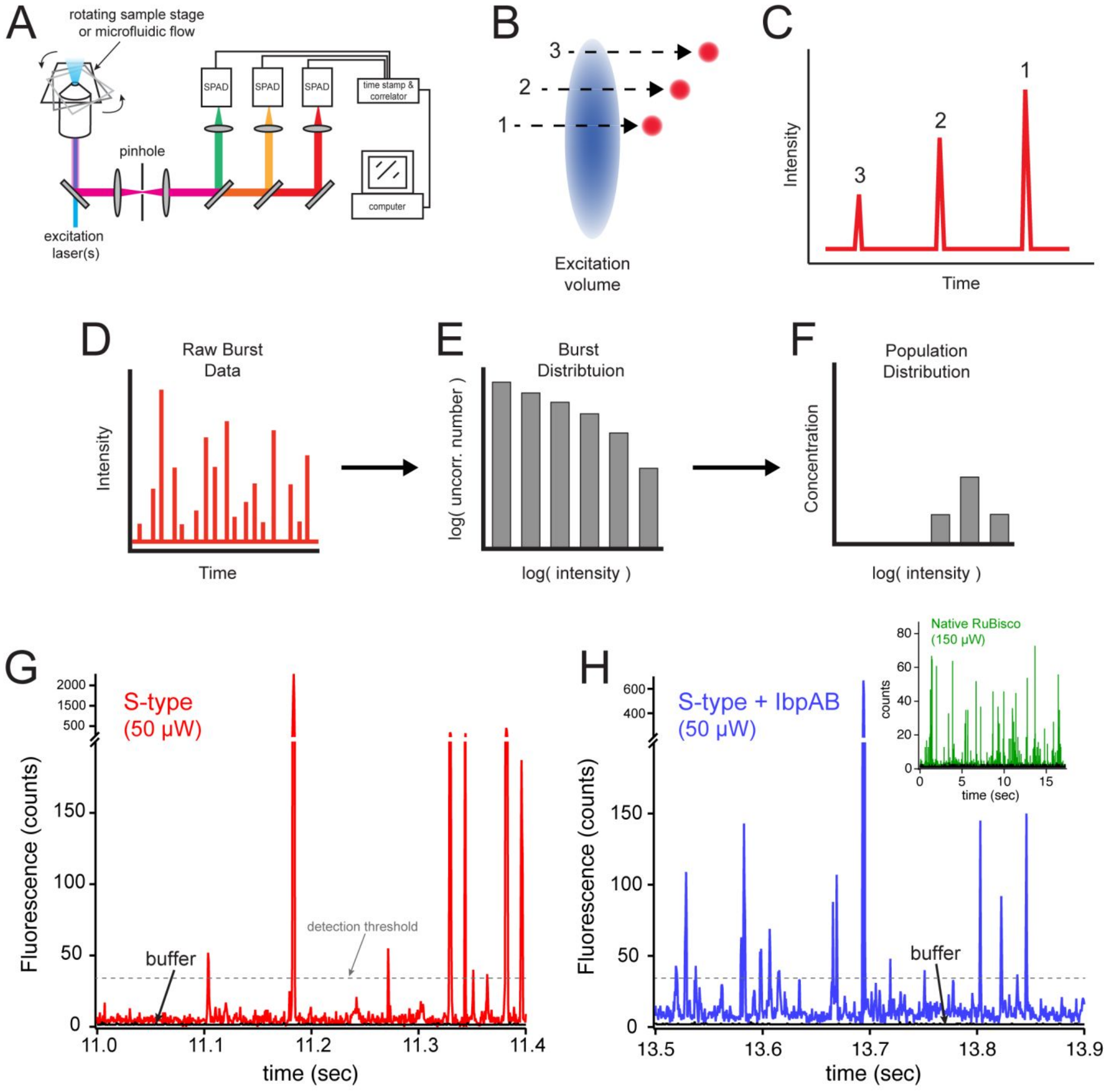

Supplemental Figure 1. Burst Analysis Spectroscopy (BAS) and example of representative raw fluorescence burst data for A647-labeled RuBisCO. (A) Schematic of a multi-channel BAS microscope, involving a system for advective sample flow, a confocal optical bench, high sensitivity photon counting detectors and precision time-tagged photon history recording. (B) A dilute (< 100 pM) nanoparticle sample is transported through the confocal excitation volume at a linear flow rate (≥ 500 μm/sec) that is faster than particle diffusion. (C) The burst intensity of single fluorescent particles is a function of both the intrinsic particle brightness and the crossing trajectory of the particle through the excitation region. (D) The raw intensity time stream can be binned (generally logarithmically) to yield a burst amplitude histogram that is equivalent to the cumulative distribution of burst events (E). Bursts in the highest intensity bin of this uncorrected distribution contain information only about the brightest

objects as they traverse the center of the detection region. The same objects crossing the confocal volume at other points, as well as smaller and dimmer particles, will only contribute to lower intensity bins. This information, in combination with knowledge of how the microscope responds to a mono-disperse population (the characteristic function) [41], can be used to reconstruct the corrected particle intensity distribution (F). Knowledge of the effective measurement volume also allows conversion of the corrected number to physical concentration. Moreover, when a functional mapping between fluorescence intensity per monomer and particle size can be determined, the intensity distribution can be converted into a particle size distribution. The graphics in A-F have been adapted from a previously published figure in ref [42]. (G and H) Representative segments of raw photon histories acquired using 50 µW (642 nm) excitation laser power for buffer (black) and S-type RuBisCO aggregates (10 nM final labeled monomer) in the absence (G; red) and presence (H; blue) of IbpAB at a mixing stoichiometry of 5:1 (IbpAB to RuBisCO monomer). (H, *inset*) Example of burst data for buffer (black) and A647-labeled native RuBisCO (50 pM dimer), acquired using 150 µW excitation laser power and a linear sample flow velocity of 500 µm/sec, (green). All photon histories are displayed at a time resolution of 0.5 msec per point. The discrimination threshold used for burst identification in all non-kinetic measurements (30 counts/0.5 msec time bin) is indicated by the gray dashed line. For disaggregation experiments, this threshold was increased (typically 75-100 counts/bin) to increase the stringency of burst identification and reject elevated background signals resulting from increased populations of very small and dim objects. Background counts, burst characteristics and noise for F-type aggregates in the presence and absence of IbpAB are essentially identical to what is observed with S-type aggregates.

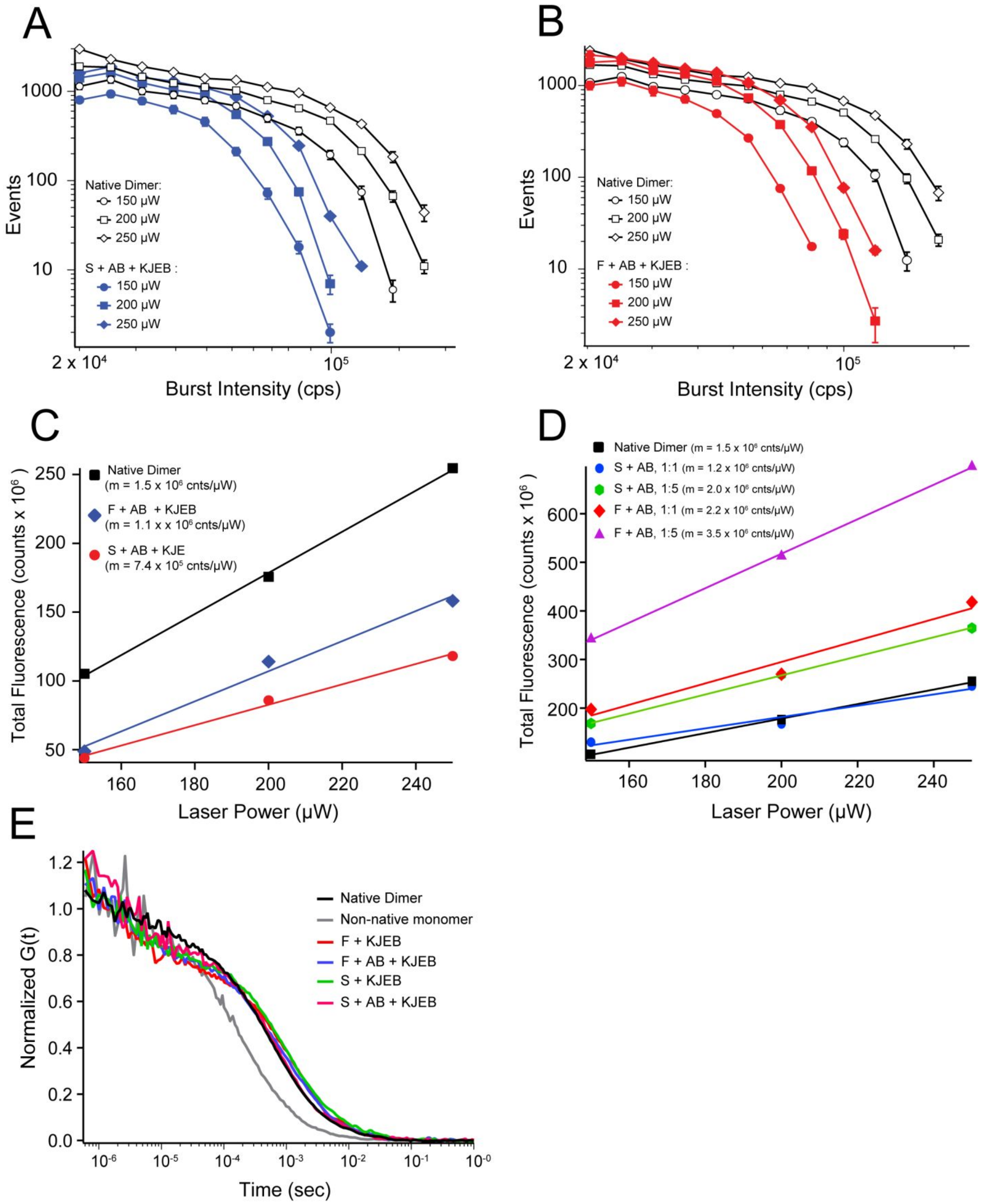

Supplemental Figure 2. Calibration of intrinsic brightness for RuBisCO-A647. (A) Photon burst distribution of single, A647-labeled native RuBisCO dimers compared to a highly diluted endpoint of a KJEB-disassembly reaction of A647-labeled RuBisCO S-type aggregates

containing IbpAB. Sample preparation and disassembly were accomplished as described in Figure 1 and 4. Each sample (< 50 pM labeled particles) flowed through the BAS microscope probe volume at a linear rate of 500 μm/sec. Samples were examined at three different excitation powers (642 nm laser) and each curve represents the average of three independent experiments, with the error bars displaying the standard deviation. The buffer background showed no detectable burst events above the minimal burst amplitude threshold employed (not shown). (B) Photon burst distribution of single, A647-labeled native RuBisCO dimers and a highly diluted disassembly endpoint of A647-labeled RuBisCO F-type aggregate particles containing IbpAB. Sample preparation and disassembly were accomplished as described in Figure 1 and 4 and data collection and analysis was accomplished as described for (A). (C) Dependence of total integrated fluorescence from single burst photon distribution curves (from A and B) on excitation laser power. (D) Dependence of total integrated fluorescence from single burst photon distribution curves (similar to A and B) for A647-labeled RuBisCO aggregate particles formed in the presence of different amounts of IbpAB: S + AB 1:1, S + AB 1:5, F + AB 1:1, and F + AB 1:5. (E) Normalized fluorescence correlation spectra (FCS) of a ~ 100 pM sample of native RuBisCO dimer, a non-native RuBisCO monomer (41), and aggregate disassembly endpoints with or without IbpAB (A647 labeling in all cases).

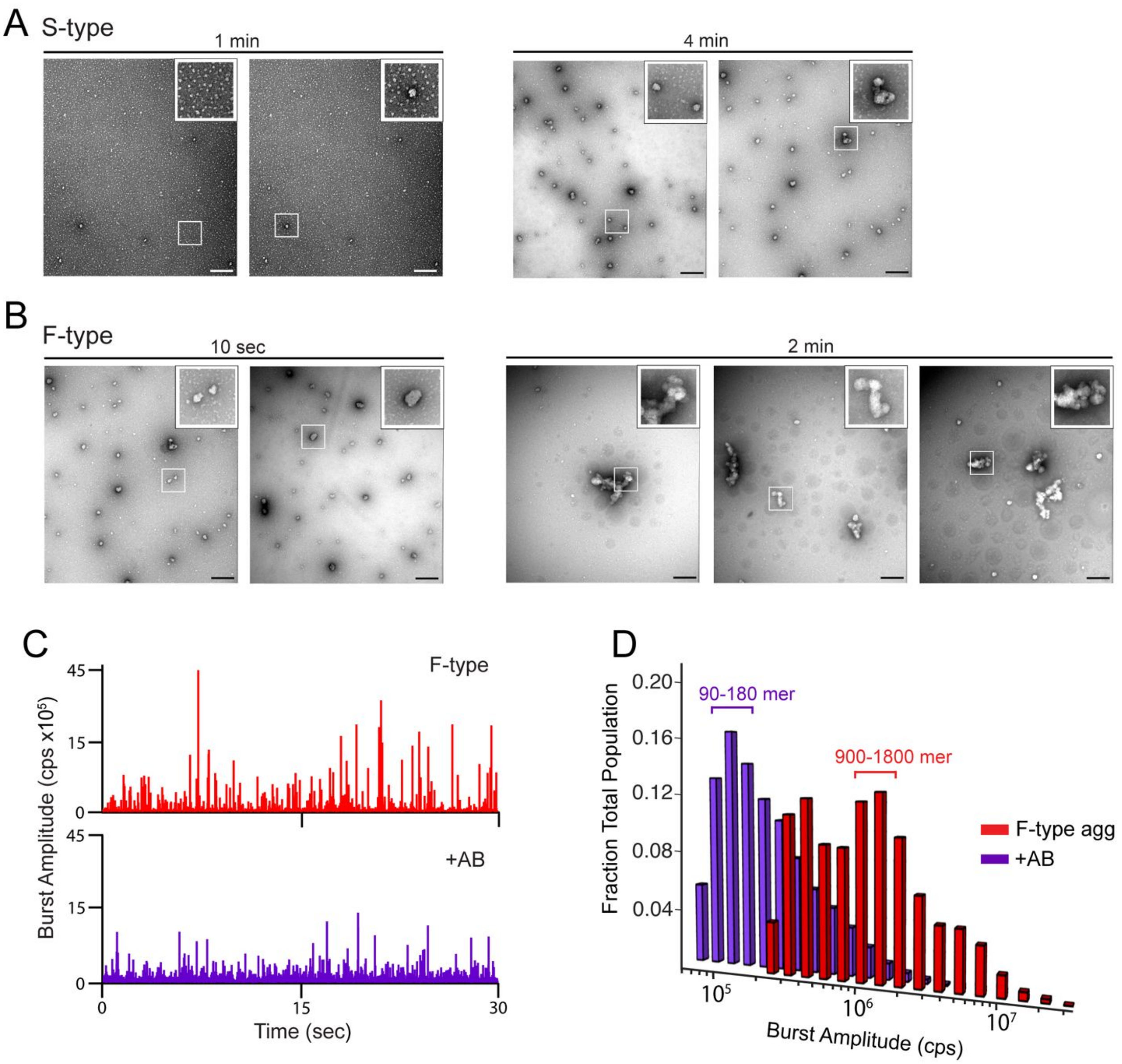

Supplemental Figure 3. RuBisCO aggregates display distinct growth morphologies and IbpAB shifts F-type aggregates to smaller particles sizes. (A) Negative stain electron microscopy images of S-type RuBisCO aggregate particles at early (1 min) and late (4 min) growth points. (B) Negative stain electron microscopy images of F-type RuBisCO aggregate particles at early (10 sec) and late (2 min) growth points. In all images, the scale bar represents 200 nm. Examples of aggregate particles are highlighted (white box) and shown at 2.5x higher magnification in the upper right inset of each image. (C) Raw photon histories showing fluorescence bursts of F-type aggregates formed in the absence (*red*) or presence (*purple*) of heat activated wild type IbpAB (5:1 IbpAB to RuBisCO monomer [10 nM]). (D) Distribution of F-type aggregate particle sizes in the presence and absence of IbpAB measured by BAS. The

approximate peak of each distribution, shown as the number of RuBisCO monomers per particle, is derived from the measured effective brightness of single Alexa647-labeled RuBisCO monomers incorporated into an aggregate particle (Figure S2 and ref [42]). Each BAS plot is a combination of n = 3, independent experimental replicates.

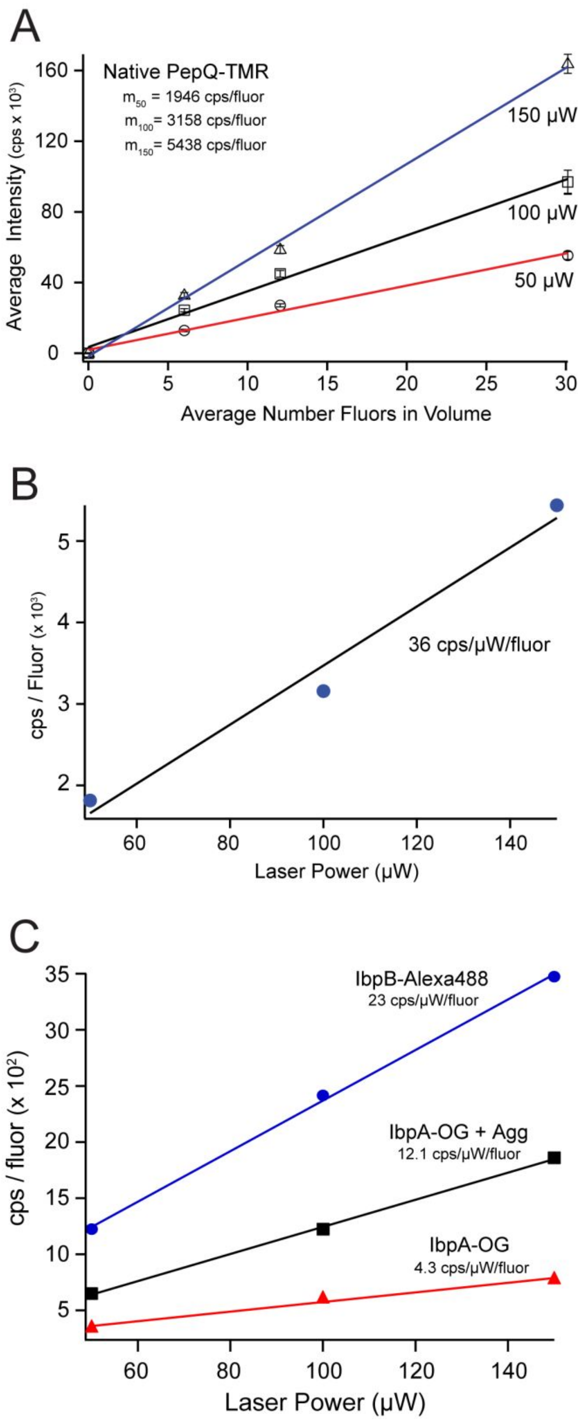

Supplemental Figure 4. Calibration of intrinsic brightness for PepQ-TMR and labeled IbpAB variants. (A) Average fluorescence of known concentrations of native PepQ-TMR at different excitation laser powers (561 nm). Error bars display the standard deviation of three independent technical replicates. Fluorescence was collected using the same BAS microscope employed for all other experiments, with the same sample configuration, but in the absence of advective flow. (B) Dependence of the average fluorescence signal of PepQ-TMR on laser power. The slope of the lines gives the intrinsic brightness of the TMR dye on a native PepQ monomer (in counts per second / µW) for the microscope configuration used in all experiments.

(C) Average fluorescence of known concentrations of IbpB-A488 and IbpA-OG, in the presence and absence of unlabeled RuBisCO aggregates (with and without S-type RuBisCO aggregates). In all cases, labeled IbpA or IbpB subunits are stoichiometrically paired with their unlabeled partner subunits. Fluorescence was collected using the same BAS microscope employed for all other experiments, with the same sample configuration, but in the absence of advective flow and following the same procedure used for panels (A) and (B). The slopes of each line give the intrinsic brightness of each probe in each context (in counts per second / μW).

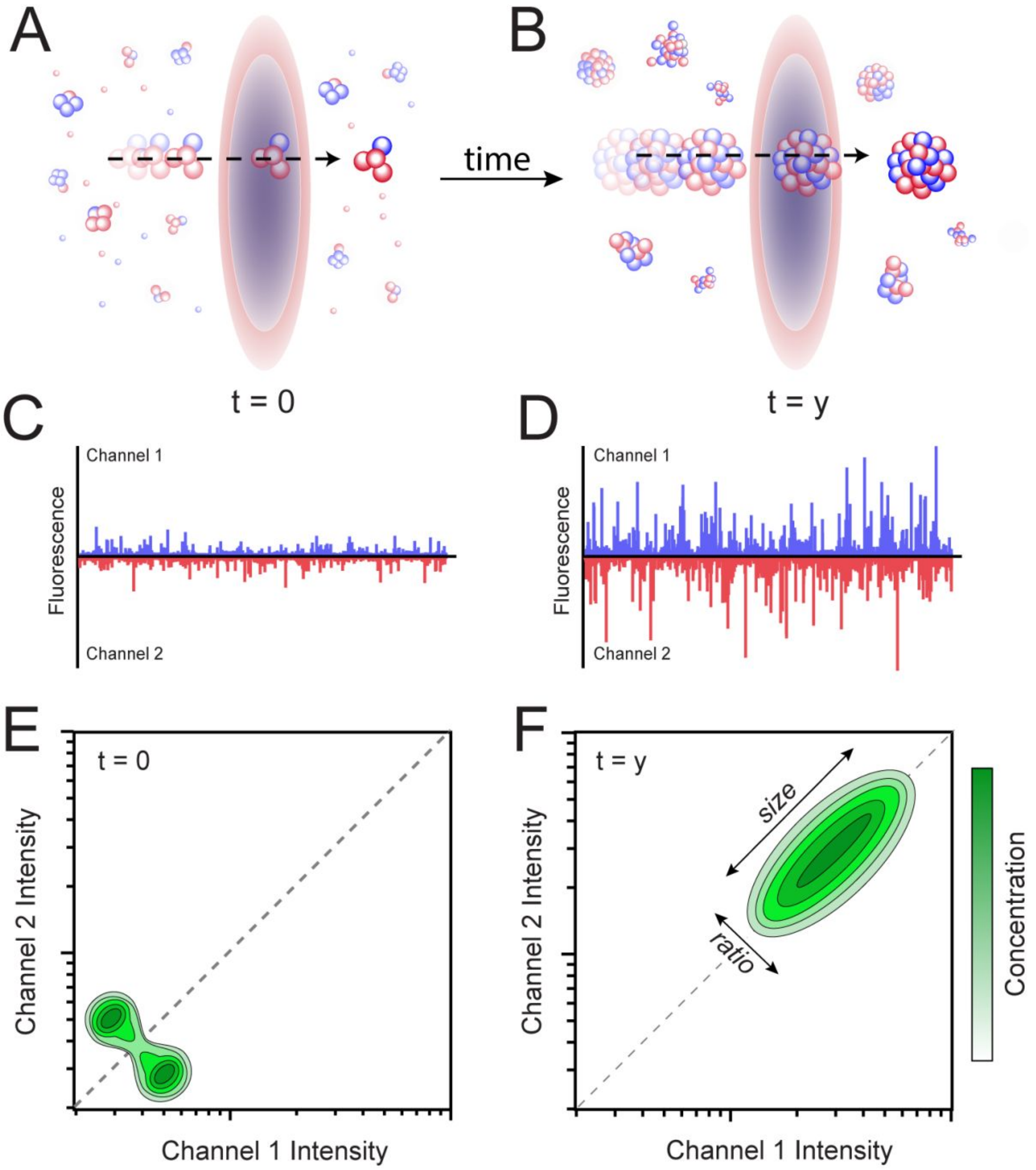

Supplemental Figure 5. Multi-color Burst Analysis Spectroscopy (MC-BAS). (A-B) A sample containing two interacting particles, which form large assemblies over time (i.e. aggregates) and are labeled with two spectrally distinct fluorescent probes, are introduced into a multi-channel BAS instrument (Figure S1). The fluorescence signal from each probe is measured using two co-aligned excitation lasers and two separate detection channels (*blue*, channel 1; *red*, channel 2) as the particles advectively flow through the observation region. (C-D) When particles assembled from differently labeled monomers cross the excitation region, they produce correlated fluorescence bursts in both channels. As with conventional BAS, the absolute burst amplitude varies in each channel as a function of both size and crossing trajectory [41]. However, particles that contain the same relative number of monomers also generate correlated bursts whose amplitude ratio is largely independent of particle size and is insensitive to crossing trajectory [42]. MC-BAS first identifies and categorizes particles by their

fluorescence intensity ratio and the burst intensity distribution from each input channel, for a given intensity ratio, is then examined by BAS and plotted in a two-dimensional heat map (E-F). Lines of fixed intensity ratio along the positive diagonal divide the resulting MC-BAS heat maps. The shape and width of each histogram captures information about the population distribution [42]. This figure has been adapted from a previously published figure in [42].

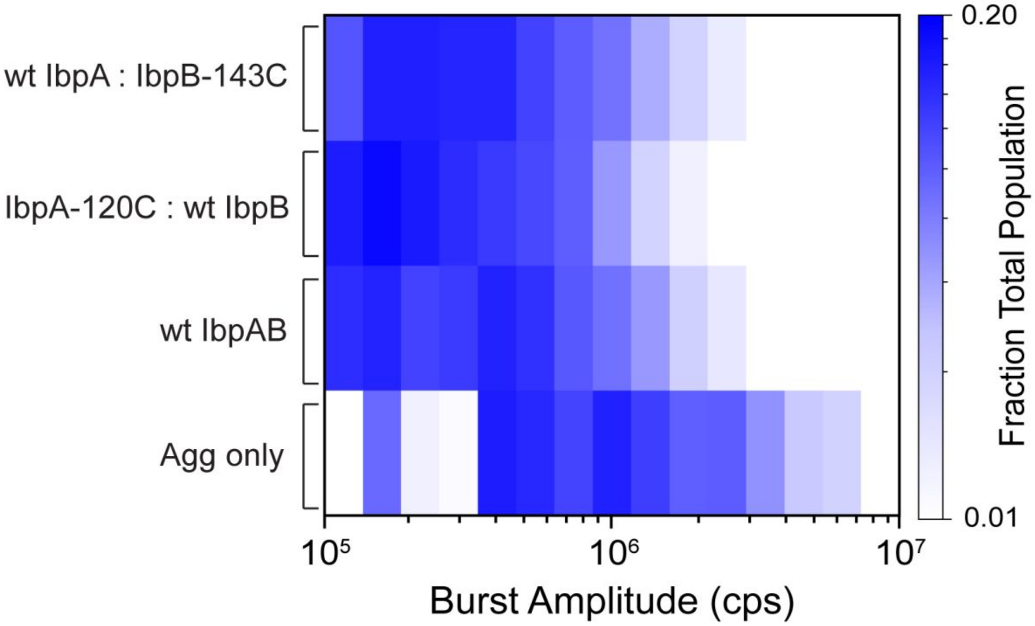

Supplemental Figure 6. Inhibition of RuBisCO aggregation is not affected by IbpA-D120C and IbpB-143C mutations. The shift in the particle size distribution of S-type RuBisCO aggregates in the presence of wild-type and mutant IbpAB dimers was examined by BAS. In all cases, 200 nM Alexa647-labeled RuBisCO monomers were mixed with either buffer alone or heat-activated IbpAB (1:1 IbpAB:RuBisCO monomers), incubated for 2 min at 4 °C followed by incubation at 23 °C for 5 min, diluted 20-fold to halt particle growth (10 nM final RuBisCO monomer) and then examined by BAS. Particle size distributions for S-type aggregates alone (Agg only), wild-type IbpAB (wt IbpAB), IbpA-120C plus wild-type IbpB (IbpA-120C:IbpB) and wild-type IbpA plus IbpB-143C (wt IbpA:IbpB-143C) are shown. The BAS plot is a combination of n = 3, independent experimental replicates for each condition.

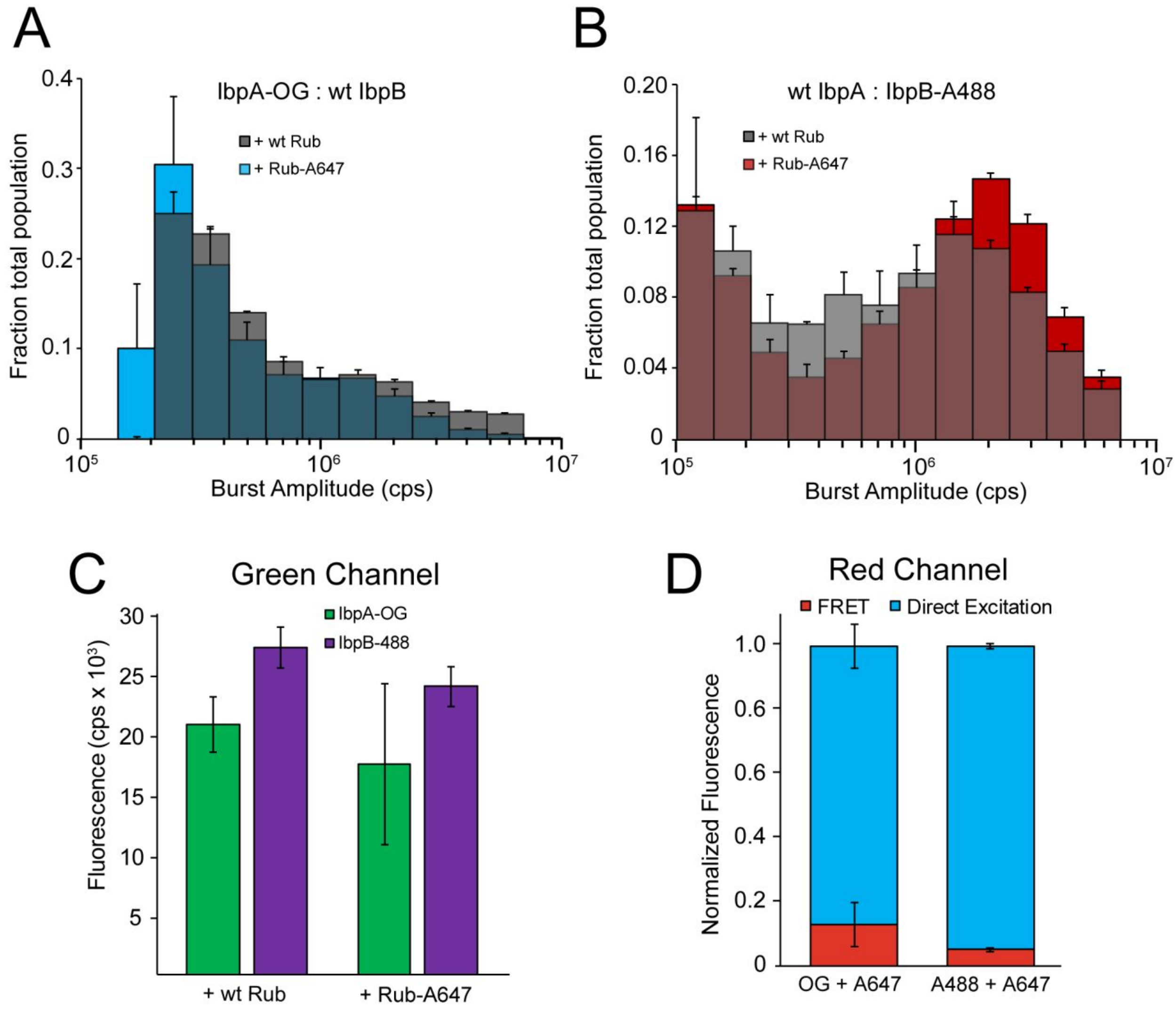

Supplemental Figure 7. Co-aggregate particle distributions formed by fluorescently labeled IbpA and IbpB are minimally impacted by either RuBisCO labeling or FRET. (A) The size distribution of fluorescent particles containing IbpA-OG (A) or IbpB-A488 (B), formed in the presence of unlabeled (wt Rub, gray) or Alexa-647-labeled RuBisCO (Rub-A647, blue or red) was examined by BAS. IbpA-OG was mixed (1:1) with wild type IbpB and wild-type IbpA was mixed (1:1) with IbpB-A488. IbpAB mixtures were then heat activated, mixed (1:1) with 200 nM RuBisCO prepared for the S-type aggregation pathway and incubated for 2 min at 4 °C, followed by incubation at 23 °C for 5 min prior to dilution (10 nM final RuBisCO monomer concentration) and BAS data collection. BAS distributions were calculated using bursts from the microscope blue detection channel only (488 nm excitation, 525 ± 18 nm emission). Because OG displays a significantly lower effective detection sensitivity than A488 under our experimental conditions (≤ 5x), the IbpA-OG distribution is truncated on the left, lower intensity side of the plot compared to the IbpB-A488 distribution. Smaller objects, which can readily be observed with A488, are lost to noise when using OG. The use of OG for labeling IbpA was a technical necessity, as A488 could not be coupled to IbpA without a dramatic loss of stability

and activity. (C) The total integrated burst signal from the blue detection channel (6 min per record) is shown for aggregate particles containing either IbpA-OG (green) or IbpB-A488 (purple), formed with either unlableld or A647-labeled RuBisCO. (D) The total integrated burst signal from the microscope red detection channel (705 ± 36 nm emission) is shown for aggregate particles formed from A647-labeled RuBisCO, in the presence of IbpAB mixtures containing either IbpA-OG or IbpB-A488. For this measurement, either the 488 nm laser alone (direct excitation of either the OG or A488 probe) or 642 nm laser alone (direct excitation of the A647 probe) was employed. The red bar illustrates the average signal in the red detection channel attributable to energy transfer (FRET) between the OG or A488 (as donors) and the A647 probe (as acceptor) during an MC-BAS experiment. The blue bar illustrates the average signal in the red detection channel attributable to direct excitation. All data shown is derived from a combination of n = 3, independent experimental replicates and error bars show ± s.d.

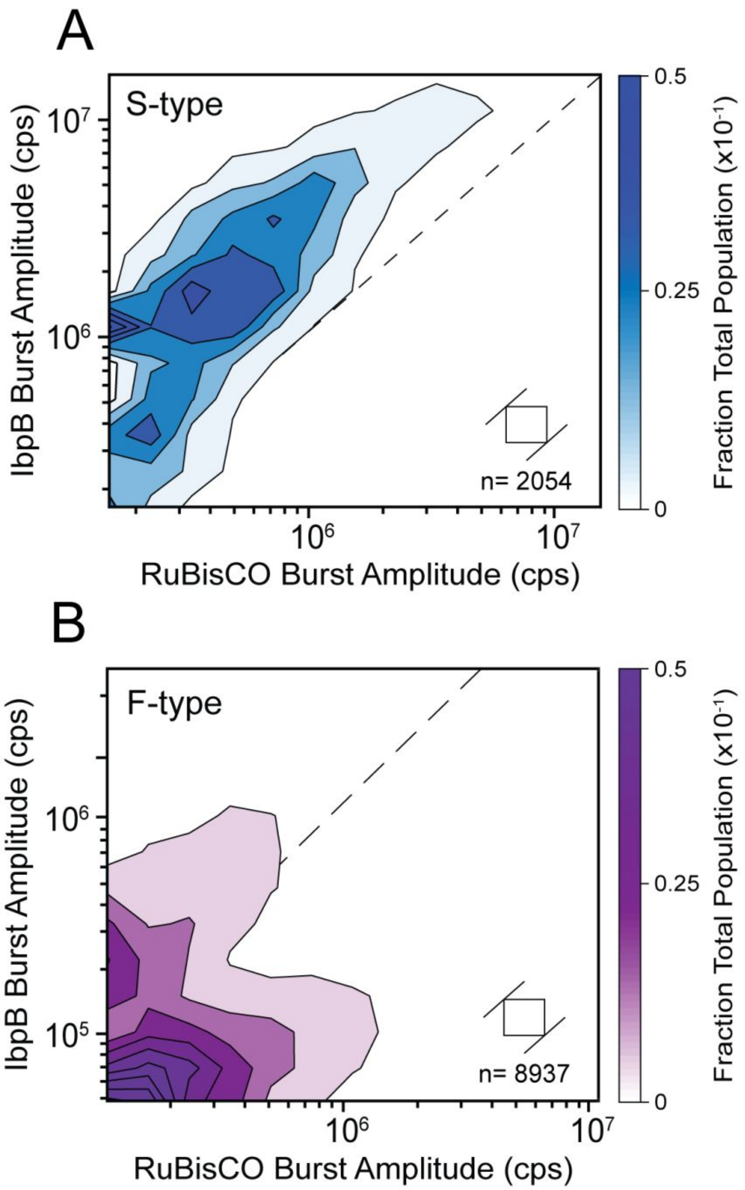

Supplemental Figure 8. The binding distribution of IbpAB on RuBisCO aggregates observed with labeled IbpB. The MC-BAS distributions for (A) S-type and (B) F-type RuBisCO aggregates bound to IbpAB using IbpB-A488. In each case, the final mixing ratio of RuBisCO monomers (10 nM) to IbpAB dimers was 1:1. The RuBisCO burst intensity is plotted on the x-axis and IbpAB burst intensity is plotted on the y-axis. The dashed diagonal line shows the experimentally determined 1:1 brightness equivalence for the RuBisCO- and IbpB-coupled A488 dye. The spread of the distributions along the positive diagonals of the plot measures the population size distribution at a given IbpAB:RuBisCO stoichiometry, while the extent of spread along the negative diagonals is proportional to the range of binding stoichiometries. Each MC-BAS plot is a combination of n = 6, independent experimental replicates. The square in each plot shows the 2D bin size prior to contour plot extrapolation and n indicates the total number of coincident burst events in data set.

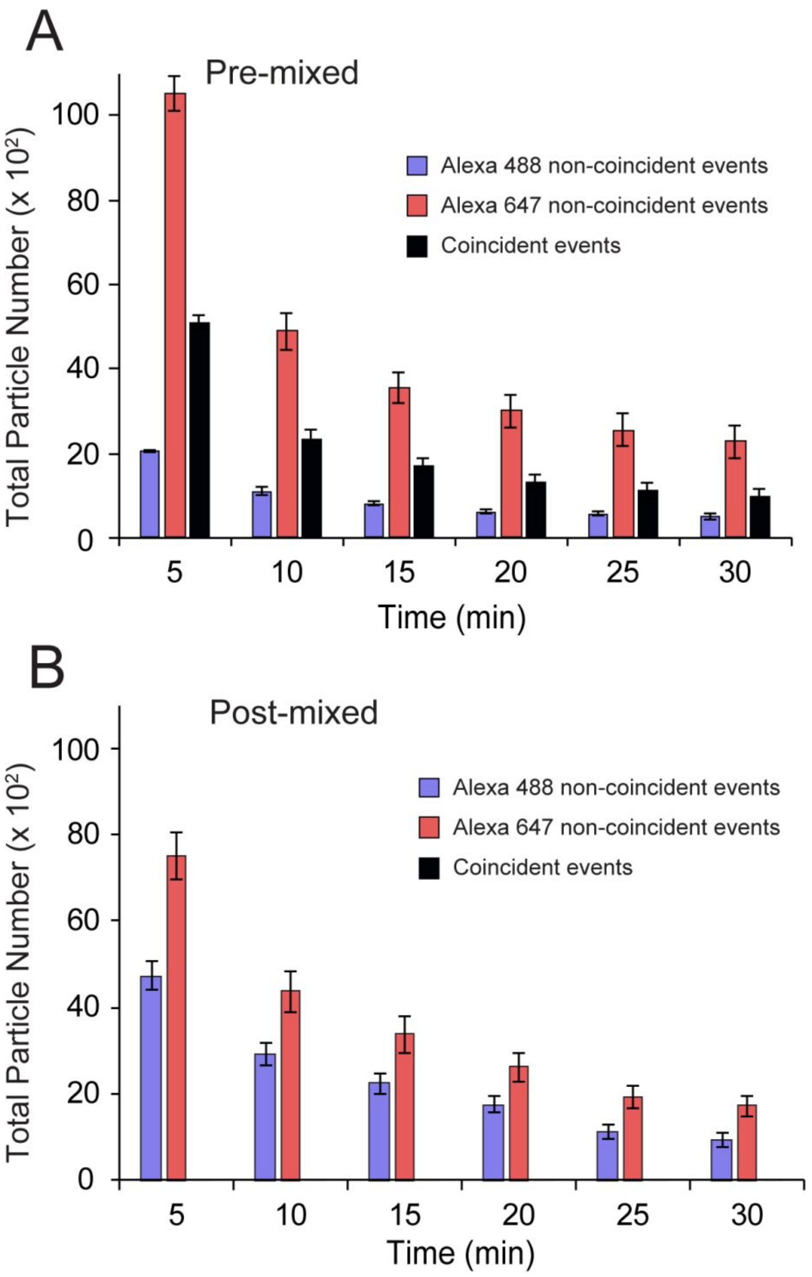

Supplemental Figure 9. IbpAB-bound RuBisCO aggregate particles do not reform during disaggregation. (A) Disassembly of co-labeled aggregate particles observed by MC-BAS as a function of time, compared with the number of particles that are only detectable in one or the other channel. S-type aggregates were formed in the presence of IbpAB at a mixing ratio of 1:1, using a 1:1 mixture of two, differently labeled RuBisCO monomers, one carrying a single Alexa488 dye and the other a single Alexa647 dye (pre-mixed). Following formation of co-labeled aggregates (10 nM final total monomer), aggregates were supplemented with 250 nM DnaK, 500 nM DnaJ, 500 nM GrpE, 50 nM ClpB, 2 mM ATP, and a creatine kinase-based ATP regeneration system. (B) Disassembly of aggregate particles by MC-BAS in which each labeled monomer was separately aggregated with IbpAB at a mixing stoichiometry of 1:1, supplemented with the KJEB system and then co-mixed after 10 sec (post-mixed). Aggregation conditions,

final total monomer and KJEB component concentrations are identical to the pre-mixed experiment in panel (A). For post-mixed samples, no particles displaying significant co-incident signals in both channels could be detected over the course of the disassembly experiment. Error bars display the standard deviation of the total number of particles detected at each time point from n = 3, independent experimental replicates.

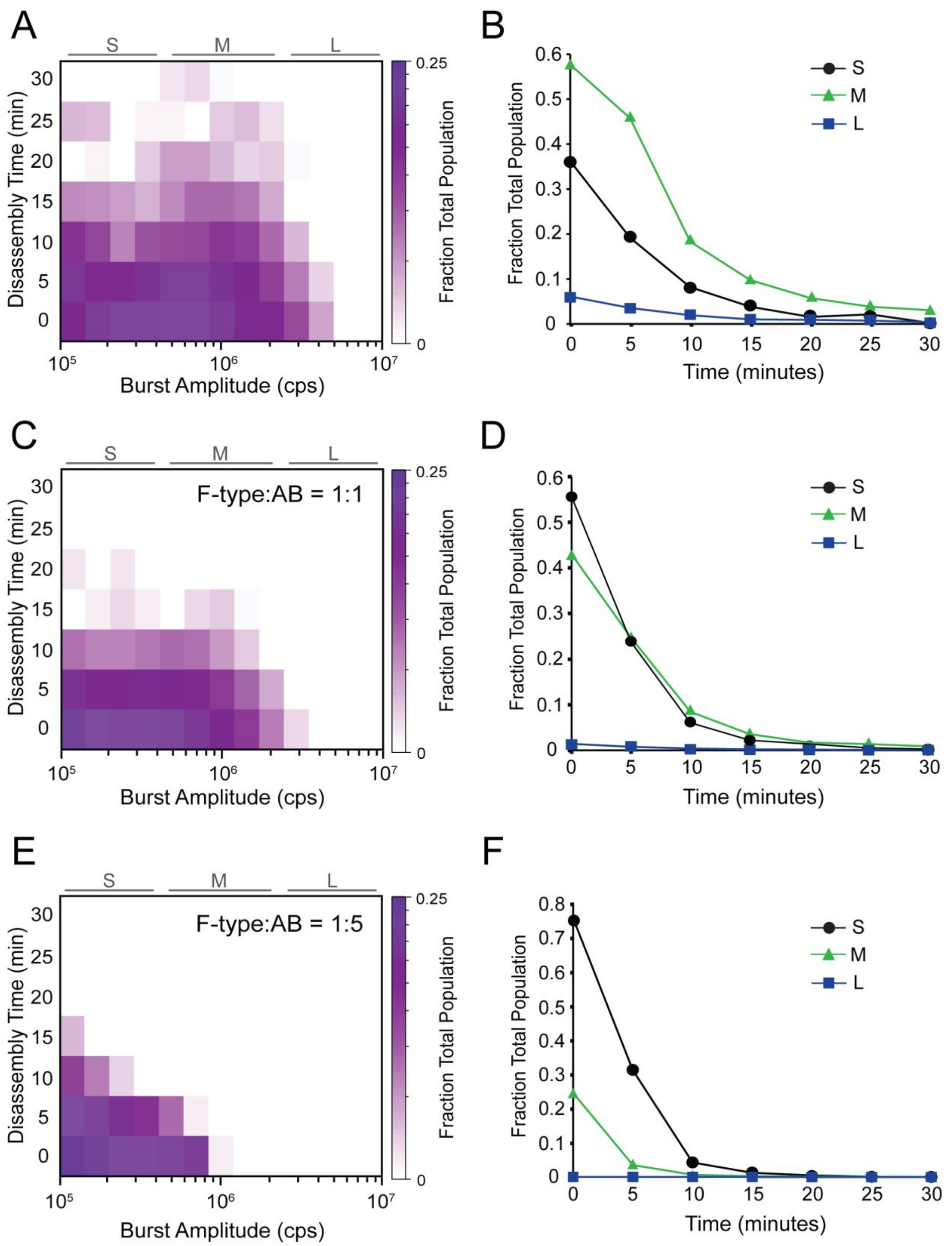

Supplemental Figure 10. IbpAB accelerates the disassembly of F-type RuBisCO aggregates by the KJEB bi-chaperone disaggregase. F-type aggregates were formed in either the absence (A) or presence of IbpAB at two different RuBisCO monomer to IbpAB ratios: 1:1 (C) and 1:5 (E). The final RuBisCO monomer concentration was 10 nM. Disaggregation was triggered by the addition of the KJEB bi-chaperone system (1 μM DnaK, 2 μM DnaJ, 2 μM GrpE and 200 nM ClpB), 2 mM ATP, and a creatine kinase-based ATP regeneration system. Samples were then loaded onto a BAS microscope and burst data was continuously acquired for 30 min.

The full experimental photon history was segmented into 5 min blocks and analysis was performed on each block. The heat maps represent a combination of three, independent experimental replicates for each aggregation condition. A zero-time measurement on each sample was collected prior to the addition of ATP. To highlight how disaggregation rates are impacted by aggregate size, the BAS heat maps were also coarsely binned into small (*S*), medium (*M*), and large (*L*) particle ranges (B, D, and F). In each case, all detected objects within a given size range were summed and plotted as a function of time following the initiation of disaggregation by KJEB.

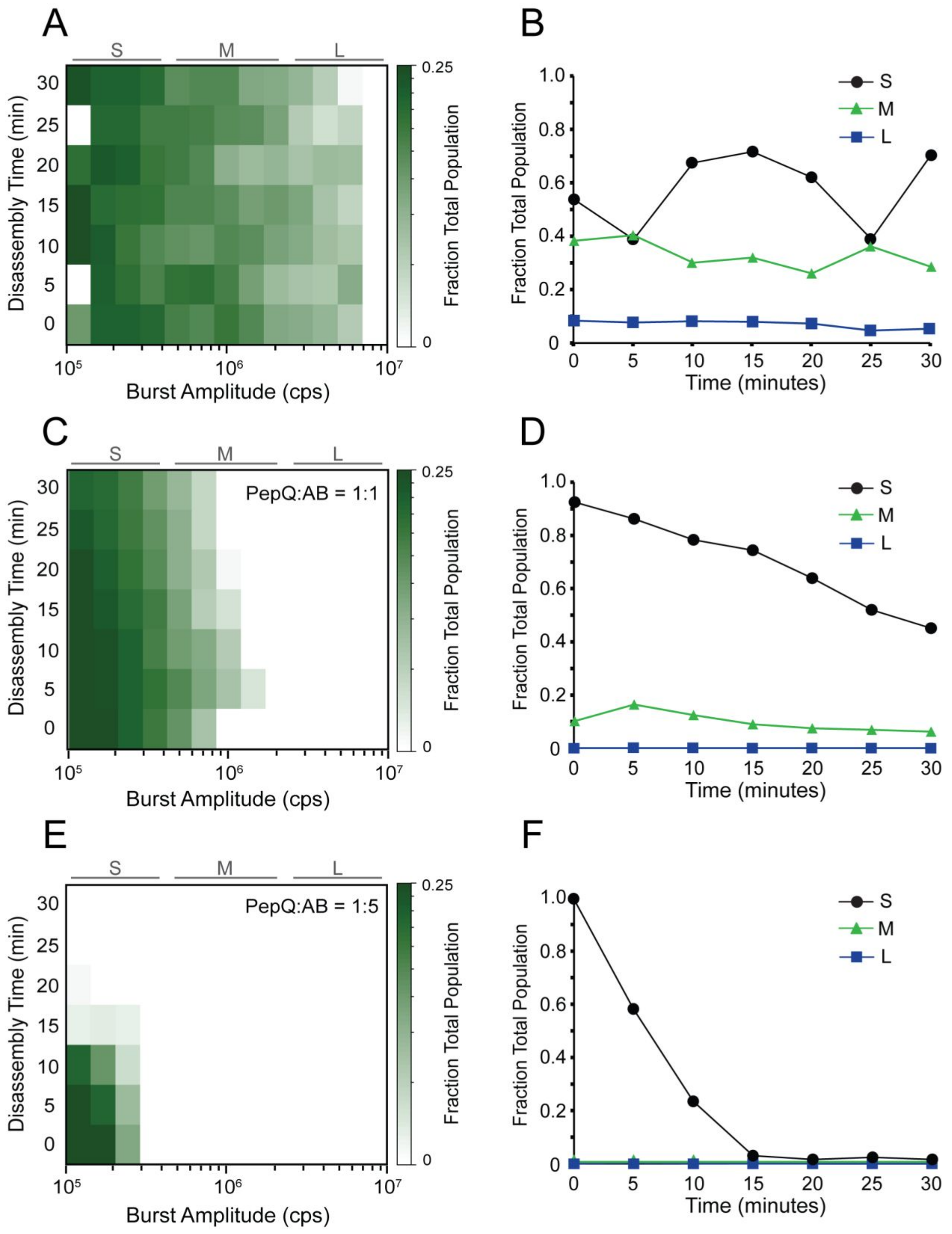

Supplemental Figure 11. IbpAB dramatically accelerates the disassembly of PepQ aggregates by the KJEB bi-chaperone disaggregase. PepQ aggregates were formed in either the absence (A) or presence of IbpAB at two different PepQ monomer to IbpAB ratios: 1:1 (C) and 1:5 (E). The final PepQ monomer concentration was 10 nM. Disaggregation was triggered by the addition of the KJEB bi-chaperone system (1 μM DnaK, 2 μM DnaJ, 2 μM GrpE and 200 nM ClpB), 2 mM ATP, and a creatine kinase-based ATP regeneration system.

Samples were then loaded into a BAS microscope and burst data was continuously acquired for 30 min. The full experimental photon history was segmented into 5 min blocks and analysis was performed on each block. The heat maps represent a combination of n = 3, independent experimental replicates for each aggregation condition. A zero-time measurement on each sample was collected prior to the addition of ATP. To highlight how disaggregation rates depended on aggregate size, the BAS heat maps were also coarsely binned into small (*S*), medium (*M*), and large (*L*) particle ranges (B, D, and F). In each case, all detected objects within a given size range were summed and plotted as a function of time following the initiation of disaggregation by KJEB.

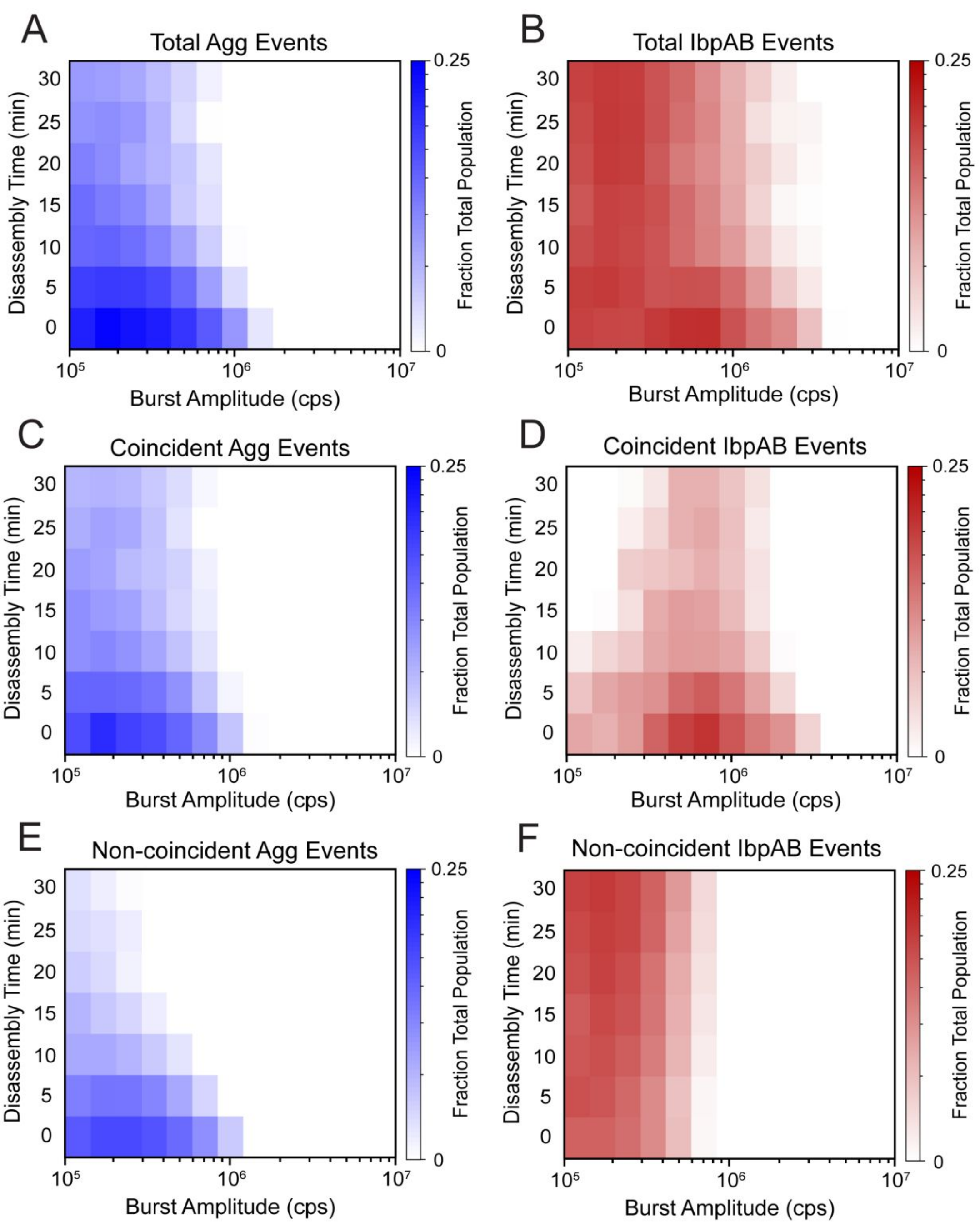

Supplemental Figure 12. Disassembly of IbpAB-bound RuBisCO aggregate particles slows when KJEB levels are reduced. The data for each channel of the MC-BAS experiment shown in Figure 6 were separately examined by standard BAS. S-type RuBisCO-A647 aggregates, formed in the presence of IbpAB (IbpA-OG) at a mixing ratio of 1:1 RuBisCO:IbpAB, were subjected to disassembly at a reduced KJEB concentration (250 nM DnaK, 500 nM DnaJ, 500 nM GrpE and 50 nM ClpB). The photon histories from the (A) RuBisCO and (B) IbpAB channels were segmented into five-minute bins and each temporal bin was then examined by BAS to give the population-resolved kinetics of total RuBisCO and IbpAB particle disassembly. The population-resolved kinetics of the coincident events only (C and D), as well as the non-coincident events only (E and F), were also examined.

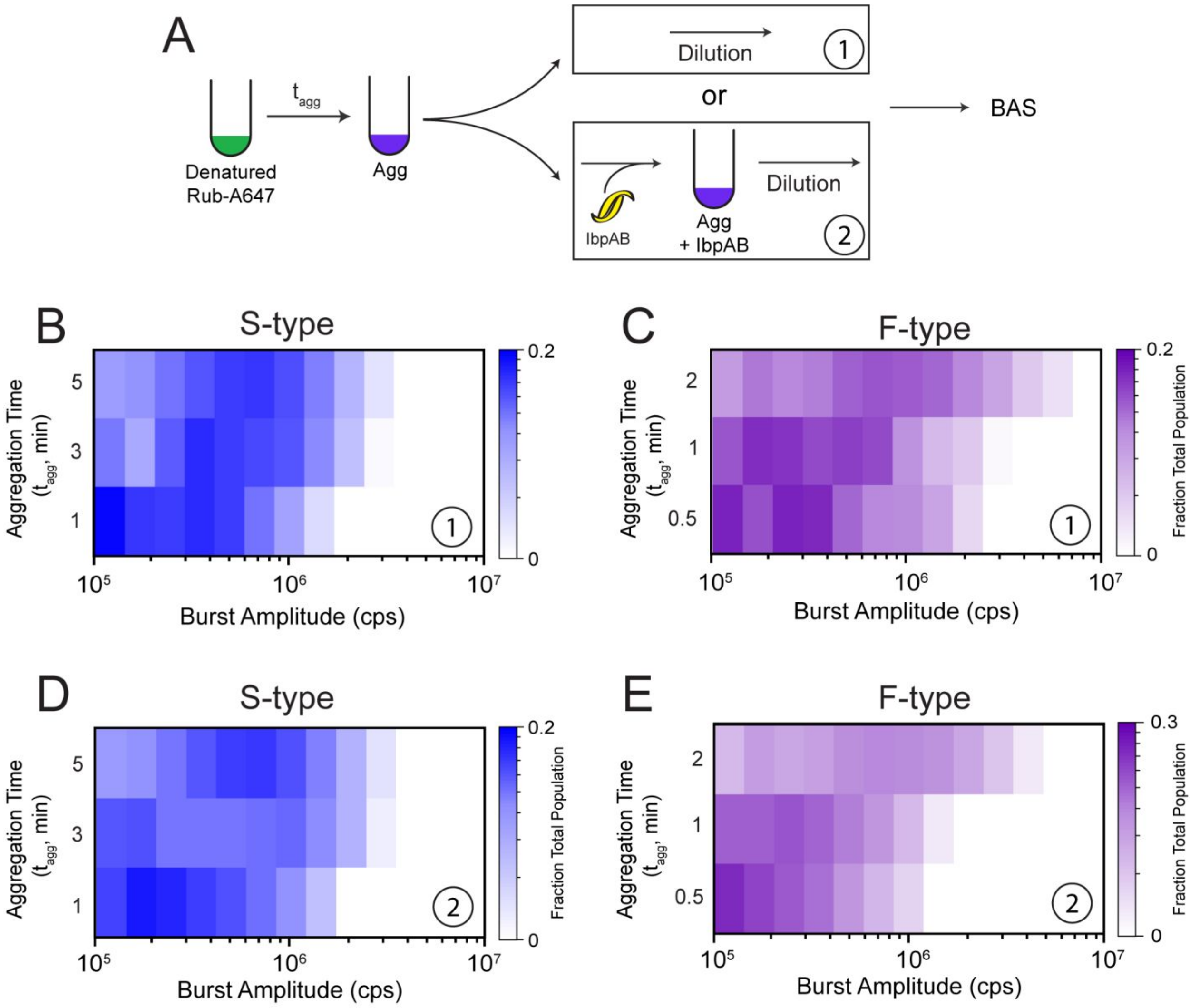

Supplemental Figure 13. Delayed addition of IbpAB halts, but does not reverse, aggregate particle growth. (A) Experimental protocol for examining the consequences of delayed IbpAB addition. At different times (t_{agg}) following the initiation of aggregation, samples were either directly diluted to halt aggregation (1) or mixed with IbpAB at 1:1 and incubated for additional time prior to dilution and measurement (2). S-type aggregate samples were incubated for a total of 5 min prior to BAS measurement and fibril-like samples were incubated for a total of 2 min. The population-resolved kinetics of particle growth for S-type (B) and F-type (C) aggregates (10 nM final RuBisCO monomer concentration in all cases) in the absence of IbpAB are shown. Delayed addition of IbpAB to either S-type (D) or F-type (E) aggregates halts particle growth at approximately the same particle distribution as dilution alone, with little or no apparent additional change in the particle size distribution over time. Each BAS plot is a combination of n = 3, independent experimental replicates.
